# The rise of weekly insulins: addressing the challenges of type 2 diabetes care in Brazil

**DOI:** 10.1186/s13098-024-01560-0

**Published:** 2025-01-15

**Authors:** André Gustavo Daher Vianna, Daniely Freitas Alves, Taís Silveira Assmann, Rosângela Roginski Réa

**Affiliations:** 1 Centro de Diabetes Curitiba, Department of Endocrine Diseases, Hospital Nossa Senhora das Graças, Curitiba, Brazil; 2 Real World Evidence, IQVIA Brazil, São Paulo, Brazil; 3 Serviço de Endocrinologia (SEMPR) do Hospital das Clínicas da Universidade Federal do Paraná (UFPR), Curitiba, Brazil; 4 Centro de Diabetes Curitiba, Rua Alcides Munhoz, 433 - 4º andar - Mercês, Curitiba, PR 80810-040 Brazil

**Keywords:** Type 2 diabetes, Once weekly insulin, Clinical inertia

## Abstract

**Background:**

Type 2 diabetes mellitus (T2D) is a global health concern with a rising prevalence, particularly in Brazil. Insulin therapy plays a crucial role in managing T2D, helping to maintain glucose and energy homeostasis. Moreover, early initiation of insulin is crucial for hyperglycemic control and prevention of chronic complications. Clinical guidelines recommend initiating insulin when other treatments fail. However, several barriers may delay its initiation, contributing to therapeutic inertia and patients’ non-adherence. These barriers include fear of hypoglycemia, lack of adherence, the need for glucose monitoring, the injection method of insulin administration, social rejection associated with the stigma of injections, fear of weight gain, a sense of therapeutic failure at initiation, and lack of experience among some healthcare professionals.

**Main body of the abstract:**

: In this context, the development of once-weekly insulin formulations could improve initial acceptance, adherence, treatment satisfaction, and consequently, the quality of life for patients. Currently, two once-weekly insulin treatments, insulin icodec and efsitora alfa, have shown promise in clinical trials, demonstrating efficacy and safety profiles similar or better than those of daily insulin therapies.

**Short conclusion:**

These once-weekly insulins have the potential to emerge as landmark achievements in the evolution of insulin therapy. This narrative review aims to evaluate the role of weekly insulins in managing T2D, providing insights into the potential benefits, challenges, and opportunities associated with a new weekly insulin therapy, specially within the Brazilian context.

## Background

Type 2 diabetes mellitus (T2D) is a global public health concern [1], affecting 536 million adults worldwide, with projections rising to 783 million by 2045 [2]. Brazil ranks sixth in T2D cases [2, 3] and third in diabetes-related health expenditure, which was USD 42.9 billion in 2021 [2]. The mortality rate has increased from 1.03 to 4.78 deaths per 100,000 from 2000 to 2021 among both males and females [1, 2].

T2D is characterized by chronic hyperglycemia due to insulin resistance and impaired secretion [1]. Insulin resistance in muscle and liver tissues impairs glucose uptake leading to hyperglycemia and T2D. By the time T2D is diagnosed, up to 50% of beta cell function may be lost [4–6].

Insulin therapy is then eventually crucial for managing T2D, benefiting approximately 100 million people globally [7, 8]. Both the American Diabetes Association (ADA) and the Brazilian Society of Diabetes (SBD) guidelines recommends initiation of insulin when the glycated hemoglobulin (HbA1c) levels exceed 10 and 9% respectively, despite other treatments [9–11].

However, insulin initiation is often delayed, increasing the risk of diabetes-related complications [12, 13]. The number and frequency of dosing are among the main barriers to insulin initiation, with a significant percentage of patients preferring good blood glucose control with fewer injections [14].

A once-weekly insulin regimen could improve treatment adherence and outcomes, reducing HbA1c levels [15–19]. Due to its stable pharmacokinetic profile, weekly insulins maintain consistent insulin levels in the bloodstream with a long and flat exposure profile. This reduces the frequency of injections from daily to weekly, simplifying the treatment regimen and reducing the burden on patients. Clinical trials have shown that weekly insulins provide comparable reductions in HbA1c levels to daily insulins. This review aims to evaluate the role of weekly insulins in managing T2D, providing insights into the potential benefits, challenges, and opportunities associated with a new weekly insulin therapy, specially within the Brazilian context.

## Challenges and disparities in managing T2D in Brazil

Brazilian patients with T2D are typically diagnosed at a mean age of 63, with nearly equal gender distribution and a slight male predominance (56%) [20]. About 85% of T2D patients are overweight or obese, highlighting the correlation between weight and T2D [21]. Chronic complications, such as coronary and cerebrovascular disease, and erectile dysfunction are highly prevalent [20].

Brazil´s ethnic diversity and socioeconomic inequalities significantly impact T2D management [22–24]. Factors like younger age, lower education level, and reliance on public service contribute to high HbA1c levels [25]. A 2019 study highlighted that economic and regional disparities in access to antidiabetic medicines are evident particularly in the Northeast, North, and Center-West regions [26–28].

Despite advances in therapy, managing T2D in Brazil is challenging, with less than half of patients achieving an HbA1c level of ≤ 7.0% [7.2 (1.3) %] after two years of consistent follow-up [25]. A meta-analysis demonstrated that globally, less than half of the people (42.8%) with diabetes achieve optimal HbA1c target, with Europe and North America presenting better results [29]. A recent real-world study conducted with T2D individuals registered in the public primary healthcare system in southern Brazil showed 30.2% of people with adequate glycemic control [30]. The 2023 Luso-Brazilian guideline recommends a tiered therapy approach based on HbA1c levels, starting with metformin and progressing to dual or triple therapy, and insulin if necessary [10].

The BrazIliaN Type 1 & 2 DiabetEs Disease Registry (BINDER) study observed that the typical treatment for T2D in Brazil includes biguanide, sulfonylureas, DPP4 inhibitors (DPP4i), SGLT2 inhibitors (SGLT2i), GLP-1 receptor agonist (GLP-1RA), and insulin. Oral medications are more frequently used than injectables, with biguanide being the most common (70.4%). Among injectable therapies, 27.3% of patients were using insulin, and 2.0% were on GLP-1RA [20]. Despite the proven cardiovascular benefits of GLP-1RAs and SGLT2i, their use is lower in Brazil compared to a overall multinational study population (18.4% vs. 21.9%, respectively), probably due to factors such as high cost and therapeutic inertia [31].

The first FDA-approved insulin analog, rapid-acting insulin lispro, was introduced in 1996, allowing faster absorption. rapid-acting insulin analogs are modified forms of insulin designed to be absorbed quickly into the bloodstream, allowing for rapid onset of action. These analogs are typically used to control postprandial (after meal) blood glucose levels. Subsequent insulin analogs, like the basal analogs glargine and detemir, improved absorption consistency and reduced nocturnal hypoglycemia. U300 and degludec offered longer duration and more stable effects. Despite these advancements, the need for convenience led to the development of weekly insulin formulations [reviewed at [32]].

In Brazil, despite the availability of various glucose lowering medications, most patients do not achieve the HbA1c target, possibly indicating clinical inertia, among other possible causes [20]. Clinical inertia, characterized by delayed therapeutic intensification, is a critical concern in T2D management [33]. Contributing factors to clinical inertia include provider-related barriers (lack of awareness or time constraints), patient-related factors (resistance to treatment changes, especially insulin initiation), and system-level challenges (such as resource limitations) [34]. Barriers to insulin therapy from a Brazilian physician’s perspective include fear for patients’ safety, limited time, and lack of proper training. From a patient’s perspective, barriers include fear and risk of potential side effects, negative perception of treatment, lifestyle impact and injection phobia. Additionally, some patients can have difficulties managing the insulin treatment, which may result in treatment discontinuation. Consequently, both physicians and patients in Brazil are often delaying insulin therapy, which has the potential to lead to clinical inertia and, consequently to poor glycemic control and increased complications [35].

Weekly insulins offer a more flexible treatment option and have the potential to enhance patient adherence to treatment. Additionally, individuals with T2D have shown a preference for a once-weekly injectable treatments [36]. Improved adherence and patient preference can be crucial factors in reducing clinical inertia and, consequently, improve diabetes control.

## Understanding weekly insulins

**Fig. 1:**
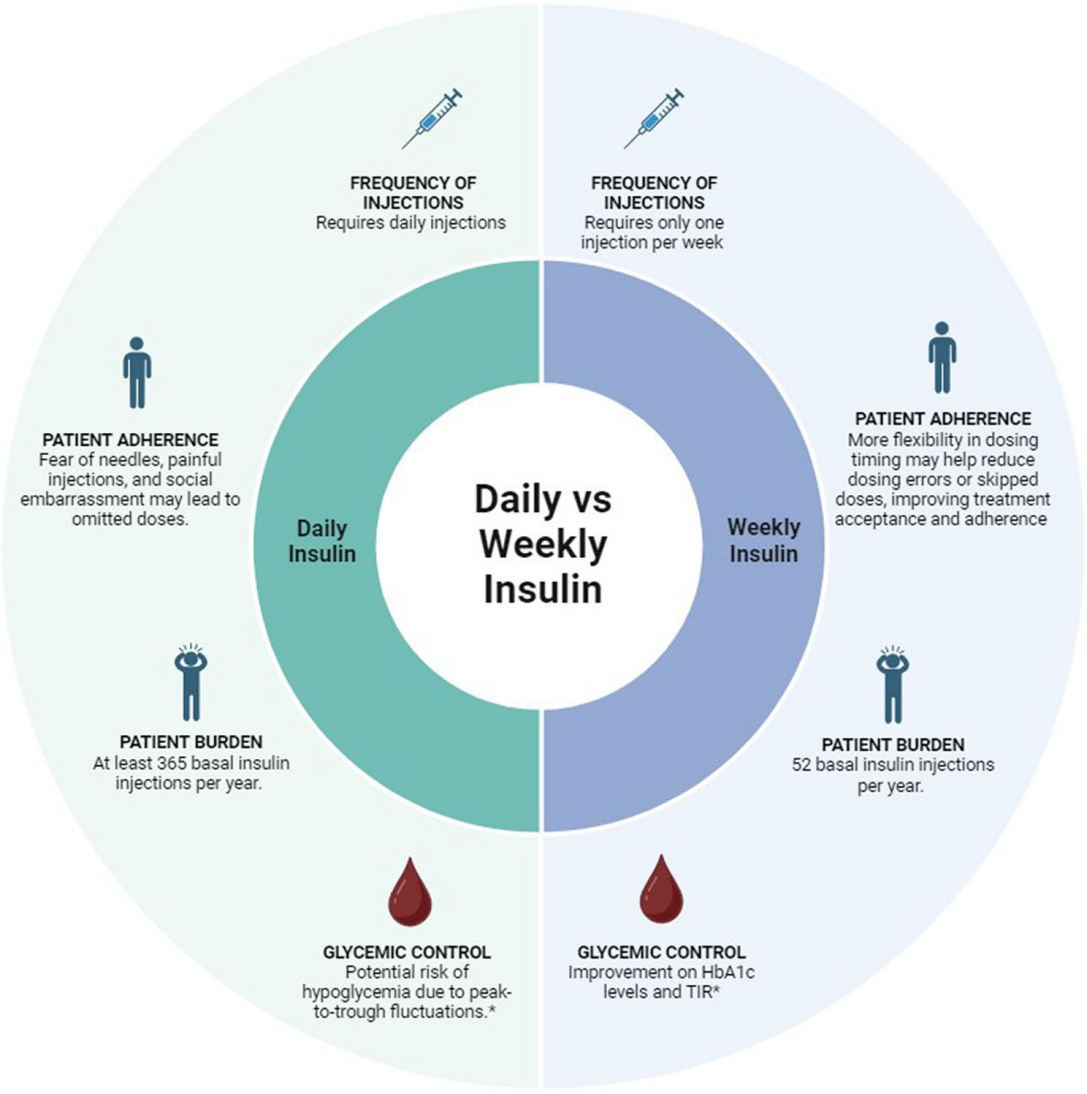
Comparison of daily and weekly insulin regimens. * The available evidence suggests a similar risk between once-weekly icodec or efisitora alfa and once-daily glargine or degludec in individuals with T2D

Weekly insulin represents an innovative approach to diabetes management. It has the potential to provide the same or increased efficacy and safety profile as once-daily insulins, while improving adherence and persistence with insulin therapy among patients with T2D [37]. A study underscored the importance of discussing both patients and healthcare providers preferences, revealing a preference for once-weekly basal insulin over current basal insulin preparations [38]. Similarly, an online survey has shown that people with T2D generally have a positive attitude towards once-weekly glucose-lowering medications, particularly among injection users (Fig. 1), increasing the chance that patients recommend the treatment to other patients [18].

T2D patients that need insulin therapy often present good glycaemic control; however, basal insulin intensification is sometimes necessary for managing specific situations such as T2D with long duration and poor glucometabolic control. Once weekly insulins provide a stable and long-acting basal insulin levels, in this way, the complementary roles of preprandial insulins or GLP-1RA and once-weekly insulins are often crucial for diabetes control, also for people with T2D [36], . Rapid-acting prandial insulins (aspart, lispro and glulisine) are new types of insulin with enhanced subcutaneous absorption, resulting in a faster onset and shorter action duration. Ultra-rapid prandial insulins (faster aspart and ultra-rapid lispro) offer even shorter onset of action, providing greater meal-time flexibility. Long-acting preprandial insulins are less common and used in specific clinical scenario, they provide extended coverage for meals [39]. Moreover, GLP-1RA work by enhancing glucose-dependent insulin secretion and used in combination with basal insulin they can also provide complementary benefits [40].

Once-daily insulin can be burdensome for patients and may lead to lower adherence due to the inconvenience and discomfort associated with frequent injections [9, 38]. On the other hand, weekly insulin requires fewer injections, potentially improving patient adherence [37]. The reduced injection burden may alleviate some of the psychological and physical discomfort associated with insulin therapy, leading to better patient outcomes [38].

## Overview of promising weekly insulins

Peptides generally have a relatively short half-life, which can pose challenges in the development of long-acting insulin formulations. To achieve a pharmacokinetic profile that lasts for at least one-week, substantial structural modifications to the insulin molecule are necessary. In this context, several different once-weekly insulin projects have been communicated. One project focuses on reducing insulin receptor affinity combined with fatty acid acylation, which is the basis for insulin icodec [41]. Two of these projects are based on fragment crystallizable (Fc) proteins [42], including efsitora alfa [43].

## Overview of icodec

Icodec insulin (Novo Nordisk) is an acylated insulin analog. Its pharmacokinetic profile is characterized by a half-life of approximately 8 days, which allows it to maintain steady-state levels with minimal fluctuations. Icodec presents three amino acid changes (TyrA14Glu, TyrB16His, and PheB25His) compared to human insulin, reducing its affinity for the insulin receptor, decreasing clearance rate through receptor-mediated process. Additionally, icodec have a 20 C fatty acid long chain attached to amino acid B29 through a hydrophilic bond, facilitating strong binding to circulating albumin, prolonging icodec half-life in the plasma. This complex acts as a reservoir, slowly releasing active insulin over time, which maintains consistent basal glucose control [19, 41]. It is based on a re-engineered version of the investigational oral basal insulin OI338, which led to a once-weekly insulin formulation [19].

Once injected, icodec is absorbed slowly due to controlled hexameric dissociation and stays binding to albumin in the bloodstream. In body tissues higher concentrations of icodec are required as it has a reduced affinity to insulin receptors [19].

Clinical trials have demonstrated icodec is well-tolerated in patients with T2D, with a half-life of 196 h and an even glucose-lowering effect throughout the week [41, 44]. Despite its low insulin receptor affinity, it requires a lower weekly clinical dose compared to insulin glargine to control blood sugar levels [44].

The phase 3 program was completed and has been submitted for regulatory review with decision anticipated for adult patients in Canada, Australia, Switzerland, China, Japan, Thailand and EMA (EU) in November 2024.

### Studies of icodec (phase 2 studies)

In the phase 2 clinical trials, icodec was compared with glargine U100 in both insulin-naive and insulin-experienced individuals with T2D (Table 1). The first published trial showed that once-weekly treatment with icodec had similar glucose-lowering efficacy and safety profile compared to once-daily glargine U100 in insulin-naive participants with T2D inadequately controlled (HbA1c level, 7.0 to 9.5%) while taking metformin with or without a dipeptidyl peptidase 4 inhibitor. The primary endpoint was change in HbA1c at week 26. Mean reductions in HbA1c were − 1.33% in the icodec group and − 1.15% in the glargine U100 group, (estimated treatment difference (ETD) [95% CI]: -0.18% [95% CI -0.38 to -0.02]; *p* = 0.08). The observed rates of hypoglycemia with severity of level 2 (blood glucose level, < 54 mg/dl) or level 3 (severe cognitive impairment) were low (icodec group, 0.53 events per patient-year; glargine group, 0.46 events per patient-year; estimated rate ratio, 1.09; 95% CI, 0.45 to 2.65). The study concluded that once-weekly treatment with insulin icodec had glucose-lowering efficacy and a safety profile similar to those of once-daily insulin glargine U100 [45] .

**Table 1:** Results from phase 2 studies

	Population	Intervention	Control	Study Design	Primary Outcome	Key Findings
**Icodec**						
Rosenstock et al. [45]	Insulin-naïve individuals with T2D	Once-weekly insulin icodec	Once daily Glargine U100	Double-blind (26 weeks of FUP, 247 participants)	Change in HbA1c at week 26	Icodec: -1.33% HbA1c reduction, Glargine U100: -1.15% HbA1c reduction, ETD [95% CI]: -0.18% [95% CI -0.38 to -0.02]; *p* = 0.08
Bajaj et al. [47]	TDM2 with previous basal insulin therapy	Once weekly icodec with an initial 100% loading dose (in which only the first dose was doubled, icodec with no loading dose	Once daily Glargine U100	Open-label (16 weeks of FUP, 154 participants)	CGM-derived TIR	Higher TIR with icodec loading dose (72.9%) vs. icodec without loading dose (66.0%) and glargine U100 (65%). No significant differences in rates of hypoglycemic events
Lingvay et al. [46]	Insulin-naïve individuals with T2D.	Once weekly icodec titrations A (prebreakfast self-measured blood glucose target 80–130 mg/dL; adjustment ± 21 units/week), B (80–130 mg/dL; ±28 units/week), or C (70–108 mg/dL; ±28 units/week)	Once-daily Glargine U100	Open-label (16 weeks of FUP, 205 participants)	Percentage time in range (TIR; 70.2–180 mg/dL)	Similar TIR across groups, higher level 2 hypoglycemic episodes in more aggressive icodec titration protocols
**Efsitora alfa**						
Frias et al. [62]	Patients with T2D previously treated with once-daily basal insulin	Weekly basal insulin Fc (Eesitora alfa)	Once-daily insulin degludec	Treat-to-target study (399 participants)	-	Similar efficacy to degludec despite higher fasting glucose targets. Lower hypoglycemia rates compared to degludec
Bue-Valleskey et al. [63]	Insulin-naive patients with T2D	Once-weekly efsitora alfa	Once-daily insulin degludec	Treat-to-target study (278 participants)	Fasting glucose target of 80–100 mg/dL	Achieved excellent glycemic control like degludec, with no concerning hypoglycemia or other safety findings

T2D: type 2 diabetes, FUP: follow-up, HbA1c: glycated hemoglobin, ETD: Estimated treatment difference, CI: confidence interval, TIR: Time in Range

A subsequent open-label randomized clinical trial (RCT) compared different icodec titration strategies with glargine U100 in insulin-naive participants with T2D over a 16-week period. Insulin was titrated weekly in four different arms, including three icodec arms (protocols A, B, C) and one glargine U100 arm. The primary outcome was the percentage of time in range (TIR; 70–180 mg/dL) during weeks 15 and 16, measured using continuous glucose monitoring. All groups achieved a similar TIR, but level 2 hypoglycemic episodes occurred at higher rates in the icodec groups with more aggressive titration protocols [groups B (80–130 mg/dL; ±28 units/week) and C (70–108 mg/dL; ±28 units/week)]. The authors concluded that icodec titration A (blood glucose target 80–130 mg/dL; adjustment ± 21 U/week) displayed the best balance between glycemic control and risk of hypoglycemia [46].

Lastly, the effects of switching from different basal insulin analogues to either icodec or glargine U100 were investigated in an open-label RCT. In this trial, insulin-treated T2D patients were randomized to icodec with an initial 100% loading dose (in which only the first dose was doubled [icodec LD]), icodec with no loading dose (icodec NLD), or glargine U100 for 16 weeks. Titration (± 28 U/week for icodec vs. ± 4 U/day for glargine) was performed weekly based on pre-breakfast self-monitored blood glucose levels, with a target of 80–130 mg/dl blood glucose in all groups. Participants receiving icodec LD achieved a higher percentage TIR than those receiving icodec NLD or those receiving glargine U100 (72.9% vs. 66.0% and 65%, respectively), with no significant differences between the groups in all grades of hypoglycemia. In conclusion, a loading dose could provide a higher percentage TIR without increases in rates of hypoglycemic events [47].

### Efficacy studies of Icodec (phase 3 - ONWARDS trials)

The ONWARDS phase 3a clinical program was designed to evaluate icodec further in individuals with either T1D or T2D across various therapeutic settings [48]. Regarding T2D, the programme comprised five trials (ONWARDS 1, 2, 3, 4, and 5) each one comparing icodec with a once-daily comparator. The main results of ONWARDS trials are shown on Table 2.

**Table 2:** Results from phase 3 studies

Trial	Design (Number of Participants, Duration)	Population	Comparator	Baseline Treatment	Main Results	Ref
**Icodec**						
ONWARDS 1	Open label (984 participants, 78 weeks)	Insulin-naive participants with T2D	Glargine U100	Any non-insulin drugs	Glycemic control was significantly better with once-weekly insulin icodec than with once-daily insulin glargine U100. TIR increase + 4.3% (CI 1.9–6.6%) *p* < 0.001	[49]
ONWARDS 2	Open label (526 participants, 26 weeks)	Insulin-treated participants with T2D	Degludec	Basal insulins ± non-insulin glucose-lowering agents	Treatment with once-weekly icodec versus once-daily degludec demonstrated non-inferiority and statistical superiority in HbA1c reduction after 26 weeks, associated with modest weight gain.	[52]
ONWARDS 3	Double-blind (588 participants, 26 weeks)	Insulin-naive participants with T2D	Degludec	Any non-insulin drugs	Once-weekly icodec demonstrated superior HbA1c reduction to once-daily degludec after 26 weeks of treatment, with no difference in weight change and a higher rate of combined level 2 or 3 hypoglycemic events in the context of less than 1 event per patient-year exposure in both groups.	[50]
ONWARDS 4	Open label (582 participants, 26 weeks)	Insulin-treated participants with T2D	Glargine U100 + aspart insulin	Multiple daily insulin injections ± non-insulin drugs	Once-weekly icodec showed similar improvements in glycemic control, with fewer basal insulin injections, lower bolus insulin dose, and with no increase in hypoglycemic rates compared with once-daily glargine U100.	[53]
ONWARDS 5	Open label (1085 participants, 52 weeks)	Insulin-naive participants with T2D	Glargine U100/300 and degludec	Any non-insulin drugs	Icodec with app showed superior HbA1c reduction and improved treatment satisfaction and compliance with similarly low hypoglycemia rates compared with OD analogues.	[51]
**Efsitora alfa**						
QWINT-1	Open label (670 participants, 52 weeks)	Insulin-naive participants with T2D	Glargine U100	At least one glucose-lowering medication	Efsitora reduced HbA1c by 1.31% compared to 1.27% for insulin glargine, resulting in an HbA1c of 6.92% and 6.96%, respectively at week 52	[65]
QWINT-2	Open label (912 participants, 52 weeks)	Insulin-naive participants with T2D	Degludec	At least one glucose-lowering medication	Efsitora reduced HbA1c by 1.26% compared to 1.17% for insulin degludec resulting in an HbA1c of 6.87% and 6.95% respectively, at week 52.	[67]
QWINT-3	Open label (986 participants, 78 weeks)	Insulin-treated participants with T2D	Degludec	Basal insulins ± up to three non-insulin drugs (except SUs)	Efsitora reduced HbA1c by 0.86% compared to 0.75% for insulin degludec resulting in an HbA1c of 6.93% and 7.03%, respectively at week 26.	[66]
QWINT-4	Open label (670 participants, 26 weeks)	Insulin-treated participants with T2D	Glargine U100	Multiple daily insulin injections	Both efsitora and insulin glargine reduced HbA1c by 1.07% resulting in an HbA1c of 7.12% and 7.11%, respectively.	[68]

TIR: Time in Range, T2D: Typer 2 diabetes mellitus, HbA1c: glycated hemoglobin, CI: confidence interval, OD: once-daily

ONWARDS 1, 3, and 5 were performed in insulin-naïve patients. The aim was to compare the effectiveness of icodec with different once-daily comparators. For ONWARDS 1 [49], the comparator was glargine U100. For ONWARDS 3 [50], it was degludec. For ONWARDS 5 [51], the comparators were pooled once-daily basal insulins (degludec, glargine U100 and U300). In these trials, icodec showed statistical superiority in reducing HbA1c from baseline to the planned end of treatment.

ONWARDS 2 and 4 were performed in insulin-experienced patients. The aim was also to compare the effectiveness of icodec with different once-daily comparators. For ONWARDS 2 [52], the comparator was degludec. For ONWARDS 4 [53], it was glargine U100. In ONWARDS 2, icodec showed statistical superiority, and in ONWARDS 4, it showed non-inferiority in reducing HbA1c from baseline to the planned end of treatment. They were all open-label studies, except ONWARDS 3, which was a double-blind, double-dummy RCT. ONWARDS 5 incorporated real-world elements, such as weekly insulin titration through a dosing app for participants and clinicians, and fewer site visits per protocol. The studies set a fasting blood glucose target of 80–130 mg/dl, with icodec weekly titration algorithm of ± 20 U based on pre-breakfast blood glucose levels from the previous 2 days plus the day the adjustment is made, in accordance with Phase 2 studies. The primary endpoint of all trials was HbA1c reduction, while secondary outcomes included TIR, time spent with blood glucose levels outside the target range, number of clinically significant or severe hypoglycemic episodes, change in fasting plasma glucose (FPG), and change in body weight.

The same pattern of weight gain was observed for icodec as with other basal insulins. In fact, a slight but statistically significant weight increase was observed when compared to degludec, but this was only observed in the ONWARDS 2 (+ 1.40 kg vs. − 0.30 kg; *p* = 0.0004) and was not seen in other phase 3 trials. Importantly, no major safety concerns related to hypoglycemia or adverse events were observed with icodec [54].

In summary, the ONWARDS trials demonstrated the potential of icodec as an effective treatment option for T2D, with similar, superior or non-inferior efficacy to once-daily basal insulin analogues and a comparable safety profile, independently of age, ethnicity, renal function, and previously use of GLP-1 analogues [55]. The trials also highlighted the benefits of icodec in terms of treatment satisfaction and compliance, further supporting its use in the management of T2D [56].

### Safety studies of icodec

In patients with T2D, continuous glucose monitoring (CGM) revealed similar duration of hypoglycemic episodes with insulin icodec and insulin glargine U100, regardless of patient experience with insulin, the titration algorithm or initial use of one-time additional or loading dose. The proportion of time spent below 54 mg/dL during a hypoglycemic episode was no longer for insulin icodec than for insulin glargine U100. Notably, no clustering of hypoglycemic episodes occurred during treatment with either insulin icodec or insulin glargine U100 [57].

Additionally, a study by Pieber et al. [58], compared the frequency of hypoglycaemia, time to hypoglycaemia, recovery from hypoglycaemia and symptomatic and counterregulatory responses to hypoglycaemia after double or tiple doses of once-weekly insulin icodec versus double or tiple doses of once-daily insulin glargine U100. The study is a randomized, single-center, open-label, two-period crossover trial, individuals with T2D. Clinically significant hypoglycemia (PG_nadir_ < 54 mg/dL) occurred similarly in both treatment arms. After a double dose, the proportion of participants with a PG_nadir_ of ≤ 45 mg/dL was comparable between the two treatments (4.7% for icodec vs. 7.1% for glargine U100; *p* = 0.63). However, after a triple dose, the glargine U100 had a higher proportion of participants with a PG_nadir_ of ≤ 45 mg/dL (2.6% for icodec vs. 25.0% for glargine U100; *p* = 0.03). Counterregulatory hormones (glucagon, adrenaline [epinephrine], noradrenaline [norepinephrine], cortisol, and growth hormone) increased during hypoglycemia induction with both insulin treatments at both doses. The hormone response was significantly higher with icodec than with glargine U100 after triple doses for adrenaline at PG_54 mg/dL (_treatment ratio 2.54 [95% CI 1.69, 3.82]; *p* < 0.001), cortisol at PG_54 mg/dL_ (treatment ratio 1.64 [95% CI 1.13, 2.38]; *p* = 0.01), and PG_nadir_ (treatment ratio 1.80 [95% CI 1.09, 2.97]; *p* = 0.02). In summary, both insulin icodec and glargine U100 induce comparable symptomatic hypoglycemia risk after double or triple dose administration, but icodec induces slightly greater endocrine responses during hypoglycemia.

In terms of hypoglycemic events, ONWARDS 3 trial demonstrated slightly higher rate of combined level 2 or 3 hypoglycemia between treatments from baseline to week 26 (0.35 in the icodec group, 0.12 in the degludec group) with less than 1 event per patient-year exposure in both groups (estimated rate ratio, 3.12 [95% CI, 1.30–7.51]; *p* = 0.01). However, no difference was observed at week 31. The odds of participants achieving HbA1c level < 7.0% with and without clinically significant or severe hypoglycemia were in favor of icodec compared to degludec [50]. A recent meta-analysis demonstrated that once-weekly insulin icodec achieved better HbA1c reduction and a higher proportion of patients reaching their HbA1c targets in comparison with once-daily basal insulin analogues [59]. The duration of overall hypoglycemic episodes (< 3.9 mmol/L or 70 mg/dL) was similar between OW icodec and OD comparators across all CGM time periods in insulin-treated participants with T2D (ONWARDS 2 and ONWARDS 4) [56, 60].

Regarding adverse events, on ONWARDS 1, ONWARDS 2, ONWARDS 4, and ONWARDS 5 no reactions at the injection site or critical issues related to medication errors were described. On ONWARDS 3, inject site reaction was described in 8.5% in icodec arm vs. 4.4% in degludec arm [50].

## Overview of efsitora alfa

Efsitora alfa (Eli Lilly and Company) is an insulin receptor agonist that is composed of a novel single-chain variant of insulin fused to a human immunoglobulin G2 (IgG2) Fc domain [61]. This fusion allowed efsitora to regulate its binding to the insulin receptor, requiring a higher local concentration for engagement; thus, providing more control of glucose uptake in the parenchyma [19].

Once injected, circulating efsitora binds to the neonatal Fc receptor (FcRn) within the endothelial cells, creating a reservoir of insulin and prolonging circulating exposure. This protection/recycling system is controlled by pH switching in the acidic endosome (∼pH 5.8) where the Fc domain/FcRn binding is favored. However, at extracellular neutral pH environment such as in the blood (pH ∼7.2), efsitora release from the FcRn is favored. This mechanism ensures a prolonged and stable insulin effect, enhancing basal glucose control [19].

Efsitora alfa is currently in advanced stages of clinical development but has not yet received approval for use. Despite the promising clinical trials results, regulatory authorities have raised concerns regarding the management of hypoglycemia and the overall benefit-risk profile, particularly for patients with T1D.

### Studies of efsitora alfa (phase 2 studies)

Efsitora’s phase 2 programme included three treat-to-target studies of which two were conducted on patients with T2D (Table 1). One study included patients with T2D previously treated with once-daily basal insulin [62], and one included insulin-naive patients with T2D [63]. In these phase 2 studies, efsitora was dosed in milligram increments from a reconstituted lyophilized powder, as the soluble insulin formulation was not available at the time of the phase 2 studies [19].

In patients previously treated with once-daily basal insulin, efsitora alfa achieved a similar efficacy compared with degludec despite higher fasting glucose targets in the efsitora alfa groups. This study included 399 participants randomized to efsitora alfa with a fasting glucose target of ≤ 140 mg/dL (group 1), efsitora alfa with a target of ≤ 120 mg/dL (group 2) or degludec with a target of ≤ 100.8 mg/dL (group 3). Titration occurred every 2 weeks in group 1, every 4 weeks in group 2 and weekly in group 3. Oral glucose-lowering medications were continued throughout the study. Higher fasting glucose targets and lower glucose variability might have contributed to lower hypoglycemia rates (both total and nocturnal) for efsitora alfa compared with degludec. These findings support continued development of efsitora alfa as a once-weekly insulin treatment for people with diabetes [62].

Among insulin-naive patients, once-weekly efsitora alfa achieved excellent glycemic control similar to degludec, with no concerning hypoglycemia or other safety findings [63]. This second phase 2 study included 278 insulin-naive participants with T2D being treated with metformin with or without a DPP4i and/ or SGLT2i, with a fasting glucose target of 80–100 mg/dL [63].

### Efficacy studies of efsitora (phase 3 - QWINT trials)

Based on the phase 2 study results, Ely Lilly has initiated a phase 3 program, entitled QWINT (Once Weekly Insulin Treatment) [64]. QWINT 1 to 4 are treat-to-target studies in people with T2D that will assess efficacy and safety of efsitora compared to a once-daily comparator (insulin degludec or glargine U100) in combination with noninsulin glucose-lowering medications. QWINT 1 compares a fixed dosing-escalation approach for once-weekly efsitora, with once-daily insulin glargine U100 as the comparator in insulin-naive patients. QWINT 2 is also studying an insulin-naive population, whereas QWINT 3 and 4 are in insulin-treated patients, the former in patients on basal insulin alone and the latter for those on basal-bolus therapy [19]. The main data regarding QWINT trials are shown in Table 2.

Positive topline results were observed from the QWINT-1-4 phase 3 clinical trials evaluating once-weekly insulin efsitora alfa in adults with T2D using insulin for the first time and those who require multiple daily insulin injections. Efsitora showed non-inferior HbA1c reduction compared to the most used daily basal insulins globally. In QWINT-1, efsitora reduced HbA1c by 1.31% compared to 1.27% for insulin glargine, resulting in HbA1c levels of 6.92% and 6.96%, respectively [65]. The safety profile of efsitora was similar insulin glargine, with a 40% lower rate of severe or clinically significant (blood glucose < 54 mg/dL) hypoglycemic events. In QWINT-3, efsitora reduced HbA1c by 0.86% compared to 0.75% for insulin degludec, resulting in HbA1c levels of 6.93% and 7.03%, respectively at week 26. Participants on efsitora or insulin degludec spent approximately two more hours per day in the target glucose range (70–180 mg/dL) compared to baseline. The safety profile of efsitora was comparable to insulin degludec, with similar rates of hypoglycemic events [66].

In both QWINT-2 and QWINT-4, efsitora was safe and well-tolerated with estimated combined rates of severe or clinically significant (blood glucose < 54 mg/dL) per patient-year of exposure of 0.58 with efsitora vs. 0.45 with insulin degludec (QWINT-2) and 6.6 with efsitora vs. 5.9 with insulin glargine (QWINT-4) [67, 68]. Additionally, in QWINT-2 the percentage of time that the glucose level was within the target range was 64.3% with efsitora and 61.2% with degludec (estimated treatment difference, 3.1% points; 95% CI, 0.1 to 6.1). No severe hypoglycemia was reported with efsitora; six episodes were reported with degludec. The incidence of adverse events was similar in both groups [69].

In summary, the QWINT programme is a comprehensive series of trials designed to evaluate the potential of efsitora alfa as a once-weekly insulin treatment for people with diabetes [64]. The results from these trials will provide valuable insights into the efficacy and safety of efsitora alfa in a wide range of therapeutic settings. These findings will be crucial in determining the role of efsitora alfa in the management of diabetes. Further studies are needed to confirm these results and to explore the long-term effects of efsitora alfa treatment.

### Combination of weekly insulin with GLP-1 receptor agonists

Given the similar frequency of injections, once-weekly basal insulins may facilitate a simplified integration with once weekly GLP-1RA. Both drugs could be administered as separate injections or as one combined fixed-dose preparation. One such fixed-ratio combination of icodec and GLP-1RA, semaglutide (IcoSema), is being evaluated in the phase 3 COMBINE clinical development program (COMBINE 1, COMBINE 2, COMBINE 3, and COMBINE 4) to evaluate the efficacy and safety of this combination therapy approach [19]. The results of these studies will provide valuable insights into the potential benefits of this combination therapy approach.

## Choosing weekly insulin

### Benefits of weekly insulin treatments

**Fig. 2:**
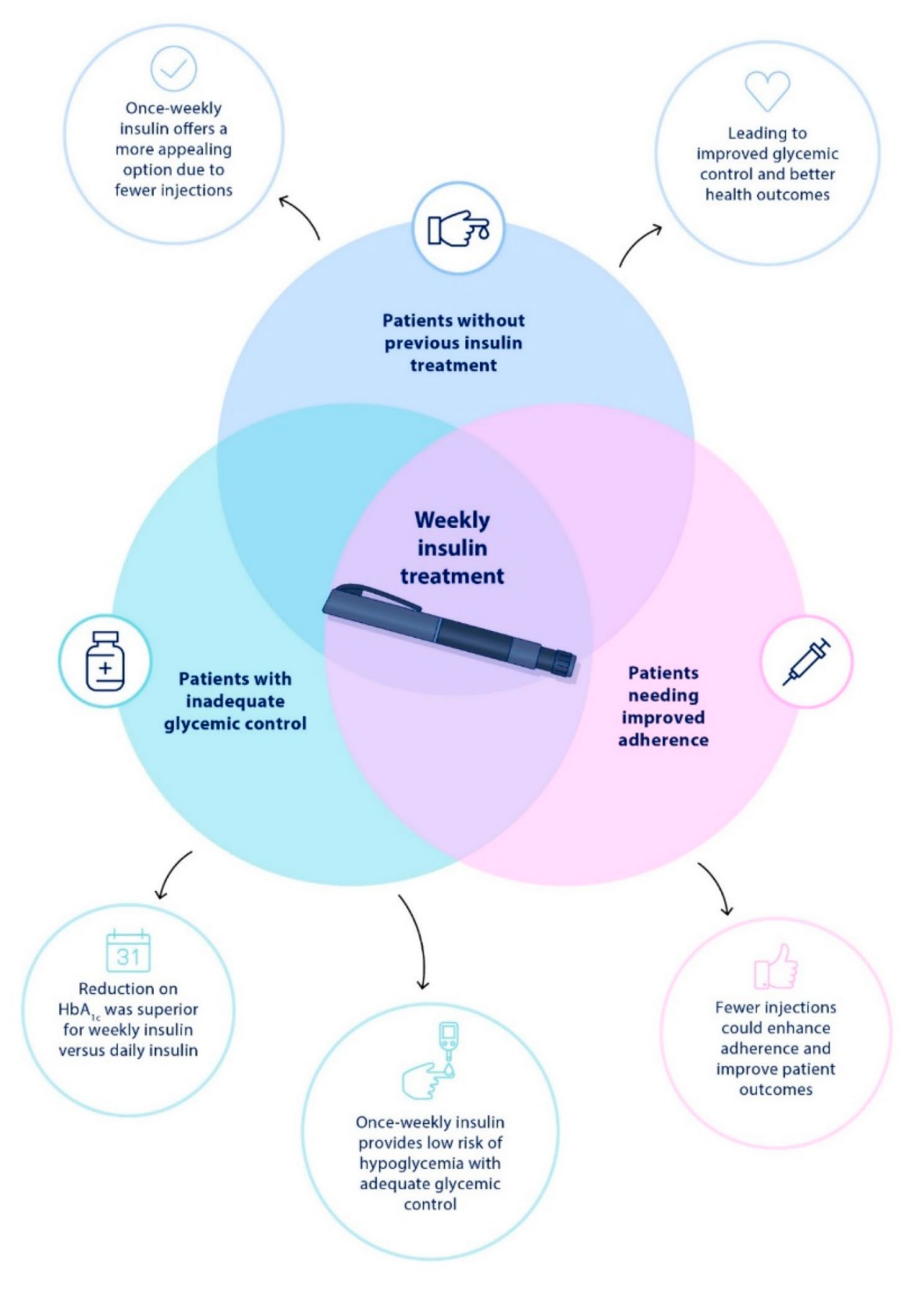
Groups of T2D patients that could benefit from weekly insulin treatment

Weekly insulin treatments can be potentially beneficial for several groups of patients with T2D, as shown on Fig. 2. These treatments simplify the regimen, reducing the number of required injections, potentially improving treatment adherence, enhancing patient quality of life, with potential savings and a positive environmental impact [17, 19].

When considering patient perspectives on using a weekly insulin regimen, several well-known patient-reported outcomes come into play. In this context, a notable enhancement was reported in the icodec groups, as measured by the Diabetes Treatment Satisfaction Questionnaire (DTSQ). Additionally, participant adherence to the treatment regimen was superior in the icodec group, assessed using the compliance domain score of the Treatment Related Impact Measure for Diabetes (TRIM-D).

In the ONWARDS 2 trial [52], patients reported significantly greater satisfaction with once-weekly insulin icodec compared to once-daily insulin degludec at 26 weeks. The reduced frequency of injections with once-weekly insulin icodec may have contributed to improved treatment satisfaction. Also in the ONWARDS 5 trial [51], the ETDs was 0.78 [CI, 0.10 to 1.47] for the DTSQ score, indicating a better treatment experience and higher satisfaction with the once-weekly insulin icodec regimen combined with the app, compared to the once-daily insulin analogues.

## Contributions to clinical practice

The role of clinicians in managing T2D is multifaceted and crucial. They are responsible for diagnosing the condition, prescribing appropriate treatments, monitoring patient progress, and adjusting treatment plans as necessary [70, 71].

**Fig. 3:**
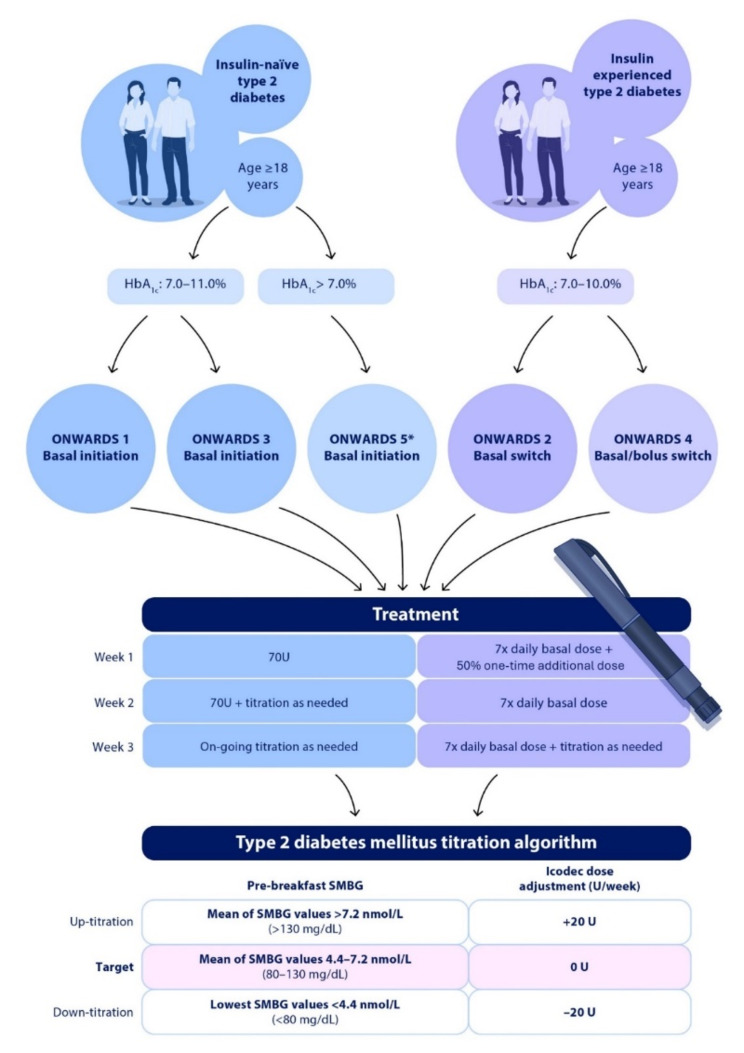
Treatment of T2D patients with icodec weekly insulin

Therefore, empowering clinicians with evidence-based insights that once-weekly insulin icodec has achieved superior glycemic control compared to once-daily basal insulin in some phase 3 trials, with no significant difference in the occurrence of hypoglycemia [72], can help them make informed decisions about the best choice of treatment for their patients in the near future (Fig. 3).

It’s important to note that individual patient characteristics and preferences should always be considered when deciding on a treatment plan.

## Expert recommendation

Considering expert recommendations, the availability of once-weekly insulin is an exciting development. For reluctant or non-adherent T2D patients, those with inadequate glycemic control, and patients in general (who wouldn’t prefer fewer injections? ), the idea of a once weekly basal insulin might be attractive.

### Hypoglycemia management

It will certainly be a challenge to educate both doctors and patients, but evidence of a relatively low risk of hypoglycemia, with episodes that do not last longer that those observed with once-daily insulins have been reassuring. Additionally, when switching from once-daily to once-weekly insulin, an once time additional dose may be necessary, which can rise concerns about overtreatment. However, comparable percentage of individuals experiencing clinically significant hypoglycemia following both double and triple doses of once-weekly icodec vs. once-daily glargine U100 were found in dedicated studies.

Despite the more stable and prolonged glucose-lowering effect of weekly insulins, hypoglycemia remains a potential risk, particularly during overnight periods or after unplanned exercise. Clinical trials with icodec and efsitora alfa have demonstrated that their risk of hypoglycemia with the use of these new insulins is comparable to that of daily basal insulins. The flatter pharmacodynamic profile reduces glycemic variability, especially the nocturnal hypoglycemia risk. However, when hypoglycemia does occur it can be manage using the same protocols as those for daily insulins, as follows:

- Immediate consumption of fast-acting carbohydrates (e.g., glucose tablets or juice) is the first line of treatment.
- Ongoing monitoring is essential due to the long-lasting effects of these insulins, as the insulin remains active for days.
- A comprehensive approach involving meal planning, exercise modifications, and possibly bolus insulin adjustments may be necessary to prevent recurring episodes. Given the prolonged duration of action, patients should be advised to remain vigilant after treating a hypoglycemic episode, as the risk of subsequent low blood sugar levels persists.

### Exercise management

Managing insulin during physical activity remains essential, especially for individuals with T1D. For patients using weekly insulins such as icodec or efsitora alfa, their long-acting nature provides a steady insulin effect throughout the week. Nevertheless, exercise can still increase the risk of hypoglycemia, depending on the intensity and duration [19]. While weekly insulins cannot be adjusted on the day of exercise due to their prolonged action, other strategies should be employed, such as:

- Adjusting bolus or mealtime insulin doses before exercise.
- Consuming additional carbohydrates based on blood glucose monitoring and the expected physical activity.
- Monitoring blood glucose levels more frequently around periods of exercise. Because these weekly insulins have a stable basal effect, exercise-induced glucose fluctuations must be managed primarily through short-acting insulin adjustments or dietary interventions.

### Emergency hospitalization

Some colleagues may be particularly concerned about emergency hospitalizations. In fact, trial participants continued once weekly icodec treatment despite hospitalization, and no substantial changes in glycemic control were observed before, during, or after hospitalization. Reported hypoglycemia rates were low, with no evidence to suggest differences before, during, or after hospitalization. Overall, the dose remained stable and uninterrupted during hospitalization. Considering how stressful and complicated hospitalizations can be this data might be reassuring regarding other issues around the use of this new insulin.

### Frequent questions about weekly insulin for the treatment of T2D

This section provides a comprehensive exploration of the clinical questions surrounding the use of weekly insulin icodec in the treatment of T2D. It addresses a variety of questions ranging from incorrect use, hospitalization data, patient concerns about dosage, to exercise management and initiation and titration strategies. Please refer to the Table 3 for detailed information.

**Table 3:** Q&A about the use of weekly insulin icodec for the treatment of T2D

Question	Answer	Reference
1. Incorrect use - If a patient forgets and applies 2x icodec, will the patient experience more hypoglycemia comparedcompared to daily insulin? Is there any study comparing double and triple doses of icodec versus glargine U100 that addresses this topic?	A similar risk of hypoglycemia is associated with double or triple doses of once-weekly icodec as with double or triple doses of once-daily glargine U100. Icodec and glargine U100 induce comparable symptomatic and moderately greater endocrine responses during hypoglycemia.	[58]
2. Missing dose - If a patient forgets to apply the icodec insulin at the scheduled day, is it possible to use it on another day? And if so, until when is it possible to apply icodec?	**Within 3 days of the missed dose**: The patient can resume their original once-weekly dosing schedule. It’s important that they monitor their fasting blood glucose levels during this time. **More than 3 days after the missed dose**: If more than 3 days have passed since the missed dose, it should still be administered as soon as possible. The once-weekly dosing schedule will then be adjusted to the day of the week when the missed dose was administered. **Maintaining the original administration day**: If the patient wishes to maintain the original day of once-weekly administration, the time between subsequent doses can be successively extended until the same administration day is obtained.	[58]
3. Hospitalization data. Is there any hospitalization data, and if so, how did icoded compare to Glargine U-100?	Among participants receiving icodec who were hospitalized during the ONWARDS trials: Most participants continued once weekly icodec treatment to trial completion despite hospitalization. Overall, the icodec dose remained stable and uninterrupted during hospitalisation. No substantial changes in glycemic control were observed before, during or after hospitalization. Reported hypoglycemia rates were low, with no evidence to suggest differences before, during or after hospitalization.	[73]
4. Did patients who used high doses of icodec complain about injection volume? Are theredata on lipodystrophy?	A meta-analysis reported that the icodec-treated group exhibited an elevated incidence of injection-site reactions. This finding was observed only in ONWARDS 3. The rates of injection-site reactions were overall low, predominantly mild in severity, and resolved promptly. Importantly, this adverse event did not lead to a reduction in prescribed drug dosages or discontinuation of the treatment regimen. It should be taken into consideration that since icodec is concentrated x7, the injection volume should be about the same as daily insulin.	[59]
5. Patients with renal and hepatic impairment - Did the studies include populations with renal and hepatic insufficiency? If so, is there an indication to adjust the dose in these populations (based on PK/PD studies in renal and hepatic dysfunction)?	No specific dose adjustment of icodec is required in individuals with renal or hepatic impairments (ONWARDS 1–5).	[74]
6. Use in patients with hypoalbuminemia - How does the use of icodec differ in patients with hypoalbuminemiay?	When considering the use of insulin icodec in patients with hypoalbuminemia, it’s important to note that the maximum serum concentration of insulin icodec at steady state is much lower than the serum albumin concentration. Each albumin molecule has multiple binding sites for insulin icodec, resulting in a significant excess of binding capacity compared to the actual insulin icodec serum concentration. As a result, even in cases of higher insulin icodec concentrations, displacement from albumin due to factors like albuminuria or competitive protein binding is minimal. Clinically, this suggests that hypoalbuminemia is unlikely to significantly affect insulin icodec activity.	[41]
7. Duration and recovery of hypoglycemia - Was the duration and recovery of hypoglycemia different with icodec compared to Glargine U-100?	**Insulin-Treated Participants with T2D (ONWARDS 2 and ONWARDS 4)** • **Switching to Once-Weekly icodec**: o TIR, TAR, and TBR were comparable between once-weekly (OW) icodec and once-daily (OD) basal insulin comparators during the switch period. o The duration of overall hypoglycemic episodes (< 3.9 mmol/L or 70 mg/dL) was similar between OW icodec and OD comparators across all CGM time periods. **Insulin-Naïve Participants with T2D (ONWARDS 1)** • **Icodec versus Glargine U100**: o TIR and TAR favored icodec over glargine U100 at weeks 22–26, 48–52, and 74–78. o TBR < 3.0 mmol/L (54 mg/dL), representing clinically significant hypoglycemia, was comparable in both groups. o CGM-derived hypoglycemia duration was similar between icodec and glargine U100.	[56, 60]
9. Glycemic behavior related to physical activities - How did glycemic control behave in relation to planned, unplanned, or suspended physical activities? What is the recommended approach for exercise management with icodec?	No evidence of increased physical activity-related hypoglycemia with once-weekly insulin icodec versus once-daily basal insulin in T2D (ONWARDS 1–5).	[75]
10. Additional dose when switching insulins -- Is additional dosing required when switching from other insulins to icodec?	PK/PD model analysis demonstrated that, among insulin-experienced individuals with T2D, switching from daily basal insulin to icodec without versus with a 50% one-time additional dose would be expected to result in a mild, transient pre-breakfast SMBG increase in the first 1–2 weeks after treatment initiation that reduced to matching levels across groups by week 4.	[47]

PD: Pharmacodynamic, PK: Pharmacokinetic, SMBG: Self-monitoring of blood glucose, TIR: Time in Range, TAR: Time Above Range, TBR: Time Below Range, OW: once-weekly, OD: once-daily

### Final considerations

Insulin therapy is a critical component in the management of T2D, but it presents several challenges that can hinder its effectiveness, such as the burden of daily injections and the risk of hypoglycemia. In Brazil, studies have shown that in general glycemic control is worse compared to other regions. The emergence of once-weekly insulins has shown promising potential in addressing these issues. Scientific data demonstrated that once-weekly insulins can improve patient adherence to treatment by reducing the frequency of injections from 365 to 52 per year, thereby enhancing convenience and reducing injection-related anxiety. Additionally, results show that once-weekly insulin are effective in managing blood glucose levels with no significant increase in hypoglycemia.

## Conclusion

In conclusion, the once-weekly insulin regimen could improve the management of T2D. Further research and real-world evidence are needed to fully understand the long-term benefits and potential challenges of this therapy. Nonetheless, the prospects of improved patient adherence and better disease management make the once-weekly insulin regimen a promising development in the fight against T2D. It is crucial to continue investing in healthcare infrastructure, patient education, and research to fully realize the benefits of these continuous improvements in the quality of life of individuals living with T2D.

## Data Availability

No datasets were generated or analysed during the current study.

## Abbreviations

ADA: American Diabetes Association
BINDER: BrazIliaN Type 1 & 2 DiabetEs Disease Registry
CGM: Continuous glucose monitoring
CI: Confidence interval
DPP4i: Dipeptidyl Peptidase-4 Inhibitors
DTSQ: Diabetes Treatment Satisfaction Questionnaire
ETD: Estimated treatment difference
Fc: Fragment crystallizable
FcRn: neonatal Fc receptor
FPG: Fasting plasma glucose
GLP-1RA: Glucagon-like peptide-1 receptor agonists
HbA1c: Glycated hemoglobulin
IgG2: Immunoglobulin G2
LD: Loading dose
NLD: Non loading dose
NPH: Neutral Protamine Hagerdorn
RCT: Randomized clinical trial
SBD: Brazilian Society of Diabetes - Sociedade Brasileira de Diabetes
SGLT2i: Sodium-glucose cotransporter-2 inhibitors
T1D: Type 1 diabetes mellitus
T2D: Type 2 diabetes mellitus
TIR: Time in range
TRIM-D: Treatment Related Impact Measure for Diabetes
